# A Crucial Role for CDC42 in Senescence-Associated Inflammation and Atherosclerosis

**DOI:** 10.1371/journal.pone.0102186

**Published:** 2014-07-24

**Authors:** Takashi K. Ito, Masataka Yokoyama, Yohko Yoshida, Aika Nojima, Hidetoshi Kassai, Kengo Oishi, Sho Okada, Daisuke Kinoshita, Yoshio Kobayashi, Marcus Fruttiger, Atsu Aiba, Tohru Minamino

**Affiliations:** 1 Department of Cardiovascular Science and Medicine, Chiba University Graduate School of Medicine, Chiba, Japan; 2 Center for Disease Biology and Integrative Medicine, Faculty of Medicine, The University of Tokyo, Tokyo, Japan; 3 Institute of Ophthalmology, University College London, London, United Kingdom; 4 Department of Cardiovascular Biology and Medicine, Niigata University Graduate School of Medical and Dental Sciences, Niigata, Japan; 5 PRESTO, Japan Science and Technology Agency, Saitama, Japan; Brigham and Women’s Hospital, Harvard Medical School, United States of America

## Abstract

Risk factors for atherosclerosis accelerate the senescence of vascular endothelial cells and promote atherogenesis by inducing vascular inflammation. A hallmark of endothelial senescence is the persistent up-regulation of pro-inflammatory genes. We identified CDC42 signaling as a mediator of chronic inflammation associated with endothelial senescence. Inhibition of CDC42 or NF-κB signaling attenuated the sustained up-regulation of pro-inflammatory genes in senescent human endothelial cells. Endothelium-specific activation of the p53/p21 pathway, a key mediator of senescence, also resulted in up-regulation of pro-inflammatory molecules in mice, which was reversed by Cdc42 deletion in endothelial cells. Likewise, endothelial-specific deletion of Cdc42 significantly attenuated chronic inflammation and plaque formation in atherosclerotic mice. While inhibition of NF-κB suppressed the pro-inflammatory responses in acute inflammation, the influence of Cdc42 deletion was less marked. Knockdown of cdc-42 significantly down-regulated pro-inflammatory gene expression and restored the shortened lifespan to normal in mutant worms with enhanced inflammation. These findings indicate that the CDC42 pathway is critically involved in senescence-associated inflammation and could be a therapeutic target for chronic inflammation in patients with age-related diseases without compromising host defenses.

## Introduction

Chronic inflammation is characterized by the long-term presence of immune cells in affected tissues and is associated with age-related diseases such as cancer, neurodegenerative disorders, and cardiovascular disease [1]. Interestingly, levels of pro-inflammatory cytokines are elevated in the endothelial cells [2] and serum [3] of older persons in the absence of disease. Thus, inflammation that accompanies the natural aging process may contribute to the onset of age-related diseases, which are responsible for most of the mortality in modern societies.

A possible link between inflammation and aging is cellular senescence [4], which is defined as irreversible growth arrest occurring after the accumulation of DNA damage response (DDR) such as activation of p53 [4], [5], and is thought to be an important anticancer mechanism [6]. There is evidence that the number of senescent cells increases in various tissues with chronological aging [6]. An important feature shared by several types of senescent cells is persistent up-regulation of inflammatory molecules such as cytokines and adhesion molecules that recruit inflammatory cells [4], [5]. The pro-inflammatory phenotype of senescent cells can be triggered by the DDR, leading to activation of NF-κB and stimulation of the production of inflammatory cytokines [4], [7], [8]. Pro-inflammatory signals emitted by senescent cells may help to prevent the development of cancer by leading to the elimination of cells with oncogenes, which have the potential to become malignant [9], [10]. Conversely, however, senescence-associated chronic inflammation could also promote tumor progression [6], [11], as well as other age-related changes such as cataract and osteoporosis [12], by disrupting cell function and tissue architecture.

Atherosclerosis is also an age-related chronic inflammatory disease [13]. In persons with atherosclerosis, chronic inflammation is mainly induced by sterile stimuli and it accelerates disease progression [13], [14]. The initial step of the atherosclerotic process involves recruitment of inflammatory monocytes to dysfunctional endothelial cells [13], [15]. Senescent endothelial cells have been suggested to represent “dysfunctional endothelial cells” since they are specifically localized in the atherosclerotic lesions of patients and share many common features, including the pro-inflammatory phenotype that can induce sterile inflammation related to atherosclerosis, [4], [5], [16]. Although senescence of endothelial cells has been implicated in the process of atherogenesis, a specific role of senescent endothelial cells in chronic inflammation associated with atherosclerosis remains uncertain due to the lack of *in vivo* models. The molecular mechanisms underlying the pro-inflammatory phenotype in senescent endothelial cells also remain unclear.

Cdc42 is a member of the Rho GTPase family, which regulates the organization, polarity, and growth of the actin cytoskeleton of cells [17]. Cdc42 has been demonstrated to be a signal transduction convergence point for intracellular signaling networks that mediates multiple signaling pathways, including tyrosine kinase receptors, heterotrimeric G-protein coupled receptors, cytokine receptors, integrins, and responses to physical and chemical stresses [17]. Aberrant activation of Cdc42 has been suggested to contribute to various pathological states, such as carcinogenesis, cardiovascular disease, diabetes, and neuronal degenerative diseases [18]. Recent evidence has also suggested a potential role of CDC42 in stem cell senescence [19] and in the aging of organisms, including humans and mice [20], [21]. In this study, we identified CDC42 as a crucial regulator of sterile inflammation induced by endothelial cell senescence. We demonstrated that deletion of *CDC42* in endothelial cells prevents chronic inflammation and plaque formation in a murine model of atherosclerosis. We also showed that knockdown of the *CDC42* pathway attenuates over-activation of innate immunity (the counterpart of inflammation) and extends the lifespan of worms, suggesting an important role of CDC42 in aging as well as in chronic inflammation.

## Results

### NF-κB regulates the expression of pro-inflammatory genes in senescent endothelial cells

**Figure 1:**
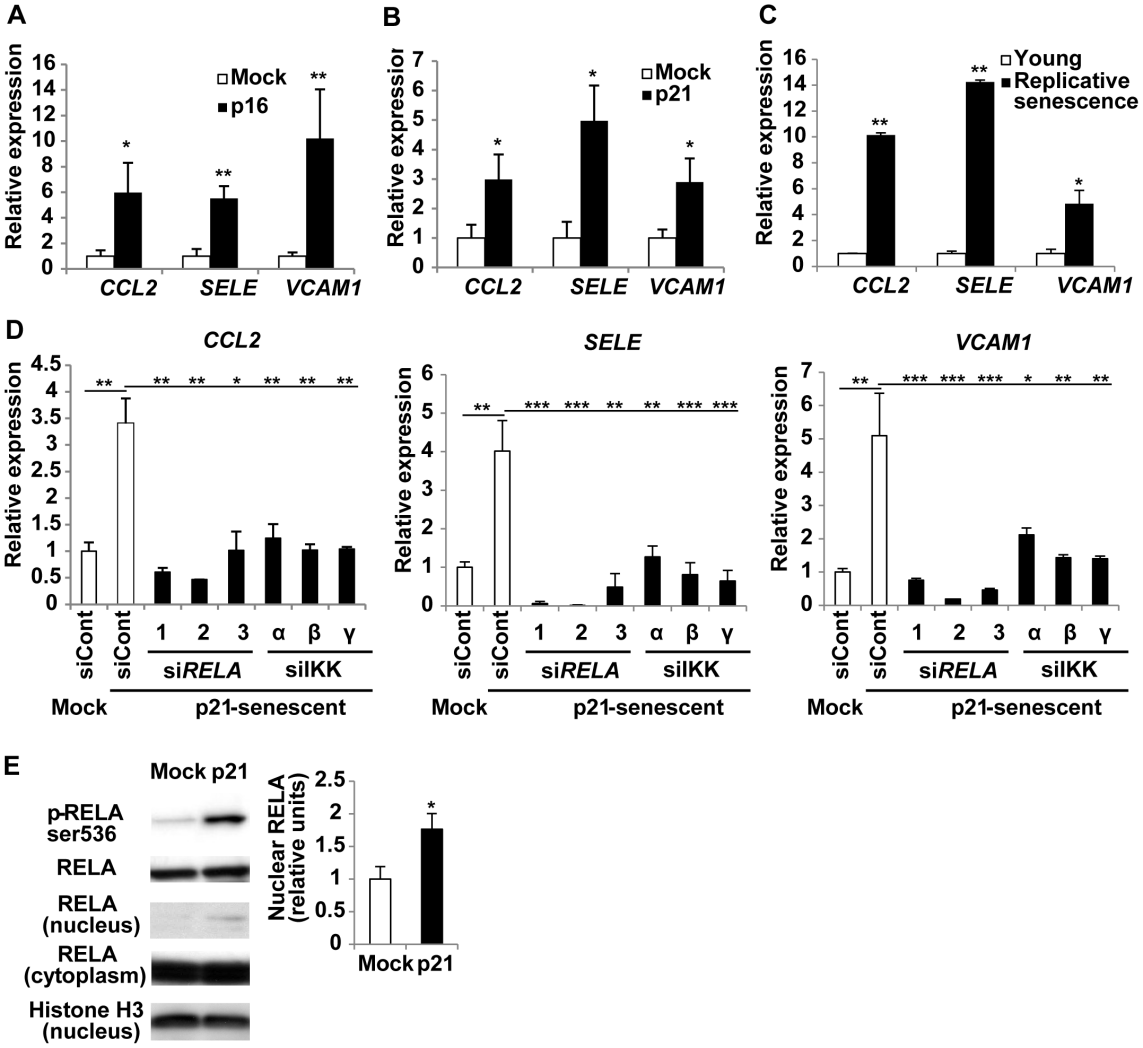
NF-κB signaling regulates pro-inflammatory gene expression in senescent endothelial cells. (A–C) Human endothelial cells were infected with an empty vector (Mock) or a retroviral vector encoding cyclin-dependent kinase inhibitor 1A (p21) or cyclin-dependent kinase inhibitor 2A (p16) to induce senescence. Expression of pro-inflammatory genes, such as the chemokine (C–C motif) ligand 2 (*CCL2*), E-selectin (*SELE*), and vascular cell adhesion molecule 1 (*VCAM1*), in senescent endothelial cells was examined by real-time PCR at 6 days after infection with the retroviral vector for p16 (A) or p21 (B) or after mock infection. Expression of pro-inflammatory genes was also examined in human endothelial cells undergoing replicative senescence (C). We defined cells with replicative senescence as cultures that did not show an increase in cell numbers and remained subconfluent for 2 weeks. n = 5. (D) Human endothelial cells were infected with an empty vector (Mock) or a retroviral vector encoding p21 to induce senescence (p21-senescent). Six days after infection, the cells were transduced with 3 sets of siRNAs for *RELA* (1–3), siRNAs for IKKs (α, β, γ subunits), or control siRNA (siCont). Expression of pro-inflammatory genes was examined by real-time PCR after 72 hours. n = 3. (E) Expression of RELA in the nuclear and cytoplasmic fractions and phospho-RELA in whole cell lysates was examined by western blotting at 6 days after retroviral infection. Samples were prepared as in Figure 1B. Histone H3 expression served as the internal control for nuclear extracts and the level of nuclear RELA relative to Histone H3 was quantified. n = 3. Data are shown as the mean ± SEM. *P<0.05, **P<0.01, ***P<0.001.

To investigate the mechanistic link between endothelial cell senescence and chronic inflammation, we introduced a retroviral vector encoding a negative regulator of the cell cycle, either cyclin-dependent kinase inhibitor 1A (p21) or cyclin-dependent kinase inhibitor 2A (p16), into human endothelial cells. Introduction of p21 or p16 led to stable cell cycle arrest with enlargement of the size of the affected cells and an increase of senescence-associated β-galactosidase activity, both of which are hallmarks of cellular senescence, and this growth arrest persisted for at least 14 days after infection (data not shown). We confirmed that there was a marked increase in the expression of p21 and p16 by human endothelial cells after infection with each vector (Figure S1A). Introduction of p21 or p16 also led to the up-regulation of pro-inflammatory cytokines and adhesion molecules relevant to atherosclerosis [15], such as chemokine (C–C motif) ligand 2 (*CCL2*; also known as monocyte chemoattractant protein-1: MCP-1), E-selectin (*SELE*), and vascular cell adhesion molecule 1 (*VCAM1*), at 6 days after infection (Figure 1A and 1B). These molecules showed similar up-regulation in human endothelial cells undergoing replicative senescence (Figure 1C). Up-regulation of pro-inflammatory genes was accompanied by senescence in all cells that we tested derived from five different individuals (Figure 1A–C and data not shown), suggesting that genetic variation among individuals does not significantly affect the inflammatory response.

We next examined the influence of knockdown of various genes by siRNA on the sustained up-regulation of pro-inflammatory genes in senescent human endothelial cells. We selected approximately 200 genes that are thought to be related to cellular senescence, metabolism, morphology, or the immune response (Table S1), and we transduced 3 siRNA sets for each gene into human endothelial cells undergoing senescence at 6 days after introduction of p21 or p16. Expression of pro-inflammatory molecules was examined by real-time PCR after 72 hours. Consistent with the results of previous studies [4], transfection of senescent endothelial cells with siRNA targeting the NF-κB component *RELA* (p65) significantly down-regulated the expression of pro-inflammatory genes to normal levels (Figure 1D and Figure S1B), while the cells remained in growth arrest (data not shown). We also found that the NF-κB pathway was significantly activated in senescent endothelial cells (Figure 1E). Indeed, knockdown of any of the three subunits of IκB kinase (IKKα, β, or γ), a positive regulator of the NF-κB pathway, normalized the expression of pro-inflammatory genes in senescent endothelial cells (Figure 1D and Figure S1B). Other studies of senescent cells have shown that DDR components, such as ATM and checkpoint kinase 2 (CHEK2), activate the transcription of pro-inflammatory genes like IL-6 and IL-8 [7], [8]. We examined six sets of siRNAs targeting *ATM* or *CHEK2* (Figure S1C), and found that inhibition of these molecules did not consistently decrease the expression of *CCL2*, *SELE*, or *VCAM1* (data not shown). The results suggest that the ATM-CHEK2 signaling pathway is not essential for regulation of these pro-inflammatory genes in senescent endothelial cells.

### CDC42 regulates pro-inflammatory gene expression in senescent endothelial cells

**Figure 2:**
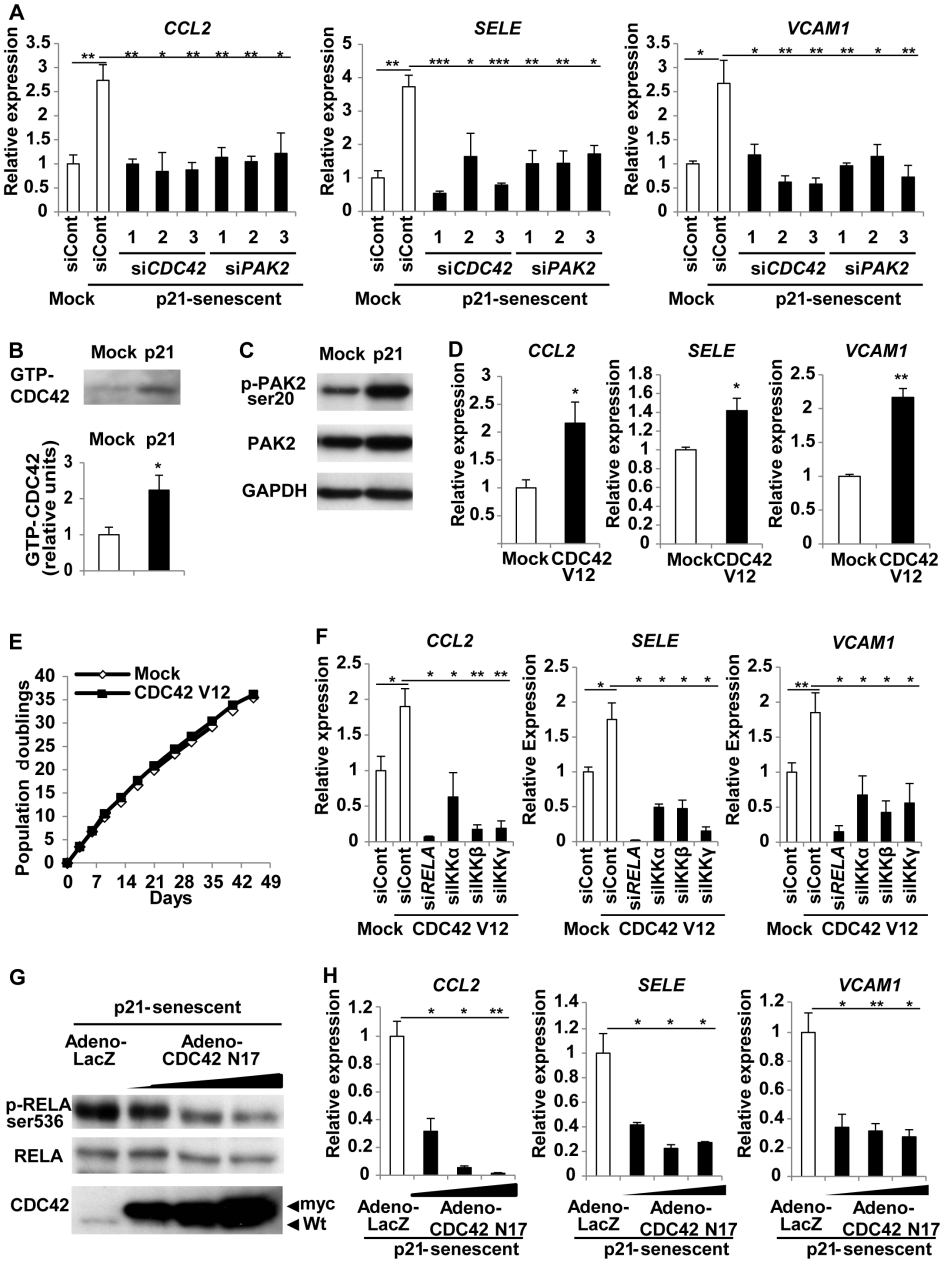
CDC42 signaling regulates pro-inflammatory gene expression in senescent endothelial cells. (A) Human endothelial cells were infected with an empty vector (Mock) or a retroviral vector encoding p21 to induce senescence (p21-senescent). Six days after infection, the cells were transduced with 3 sets of siRNAs for *CDC42*, siRNAs for *PAK2*, or control siRNA (siCont). Expression of pro-inflammatory genes was examined by real-time PCR after 72 hours. n = 3. (B) Human endothelial cells were infected with an empty vector (Mock) or a retroviral vector encoding p21 to induce senescence (p21) and were harvested at 6 days after infection. The pull-down assay for active CDC42 (GTP-CDC42) was performed as described in Methods. The graph indicates the relative level of active CDC42. n = 3. (C) Expression of phopho-PAK2 and total PAK2 was examined by western blotting in human endothelial cells prepared as in Figure 2B. (D) Human endothelial cells were infected with an empty vector (Mock) or a retroviral vector encoding active CDC42 (CDC42 V12), and expression of pro-inflammatory genes was examined by real-time PCR at 6 days after infection. n = 3. (E) Proliferation of endothelial cells infected with a retroviral vector encoding active CDC42 (CDC42 V12) or an empty vector (Mock). n = 3. (F) Human endothelial cells were infected with an empty vector (Mock) or a retroviral vector encoding active CDC42 (CDC42 V12). Six days after infection, the cells were transduced with 3 sets of siRNAs for *RELA*, siRNA for IKKs, or control siRNA (siCont). Expression of pro-inflammatory genes was examined by real-time PCR after 72 hours. n = 3. (G) Human endothelial cells were infected with a retroviral vector encoding p21 to induce senescence (p21-senescent). Six days after retroviral infection, the cells were infected with an adenoviral vector encoding a dominant-negative form of myc-tagged CDC42 (CDC42 N17) or LacZ. Expression of phospho-RELA (Ser536) and total RELA were examined by western blotting at 48 hours after adenoviral infection. (H) Expression of pro-inflammatory genes in endothelial cells prepared as in Figure 2G was examined by real-time PCR. n = 3. Data are shown as the mean ± SEM. *P<0.05, **P<0.01, ***P<0.001.

Among genes unrelated to the canonical NF-κB pathway, we found that knockdown of the CDC42 pathway significantly reduced the senescence-associated increase of pro-inflammatory molecules (Figure 2A). CDC42 belongs to the small Rho GTPase family that regulates the organization of the cytoskeleton and membrane in relation to cell polarity, proliferation, and motility [18]. Knockdown of *CDC42* or *PAK2* (one of the downstream kinases) suppressed the up-regulation of inflammatory genes (Figure 2A and Figure S1B). siRNAs targeting these genes changed the cell morphologies of treated cells. However, the changes of the cell shape were inconsistent among the 3 siRNAs targeting the same gene while the cells remained growth arrested in all the siRNA-treated senescent cells (data not shown) suggesting that the morphological changes did not reflect the escape from cellular senescence. Knockdown of other family members such as Rho (*RHOA* and *RAC1*) and PAK (*PAK1*, *3* and *4*) had less effect than that of *CDC42* or *PAK2* (data not shown). Like the NF-κB pathway, both CDC42 and PAK2 were activated as cells underwent senescence (Figure 2B and 2C). To investigate the potential relationship between CDC42 and NF-κB, we introduced an active form of *CDC42* (*CDC42* V12) [22] into normal endothelial cells (Figure 2D–F) by retroviral infection. Activation of CDC42 led to a significant increase of the expression of *CCL2*, *SELE,* and *VCAM1* (Figure 2D). However, there was no alteration of the replicative lifespan of the cells (Figure 2E), suggesting that CDC42-induced up-regulation of pro-inflammatory genes was unrelated to cell cycle regulation. In addition, knockdown of the NF-κB pathway markedly inhibited the up-regulation of pro-inflammatory gene expression induced by active CDC42 (Figure 2F and Figure S2). Moreover, introduction of a dominant-negative form of CDC42 (CDC42 N17) [22] into p21-induced senescent endothelial cells significantly down-regulated NF-κB activity as well as the expression of pro-inflammatory genes (Figure 2G and H). These findings provide evidence that CDC42 up-regulates the expression of pro-inflammatory molecules in endothelial cells by activating the NF-κB pathway.

### CDC42 regulates senescence-associated inflammation more specifically than NFκB

**Figure 3:**
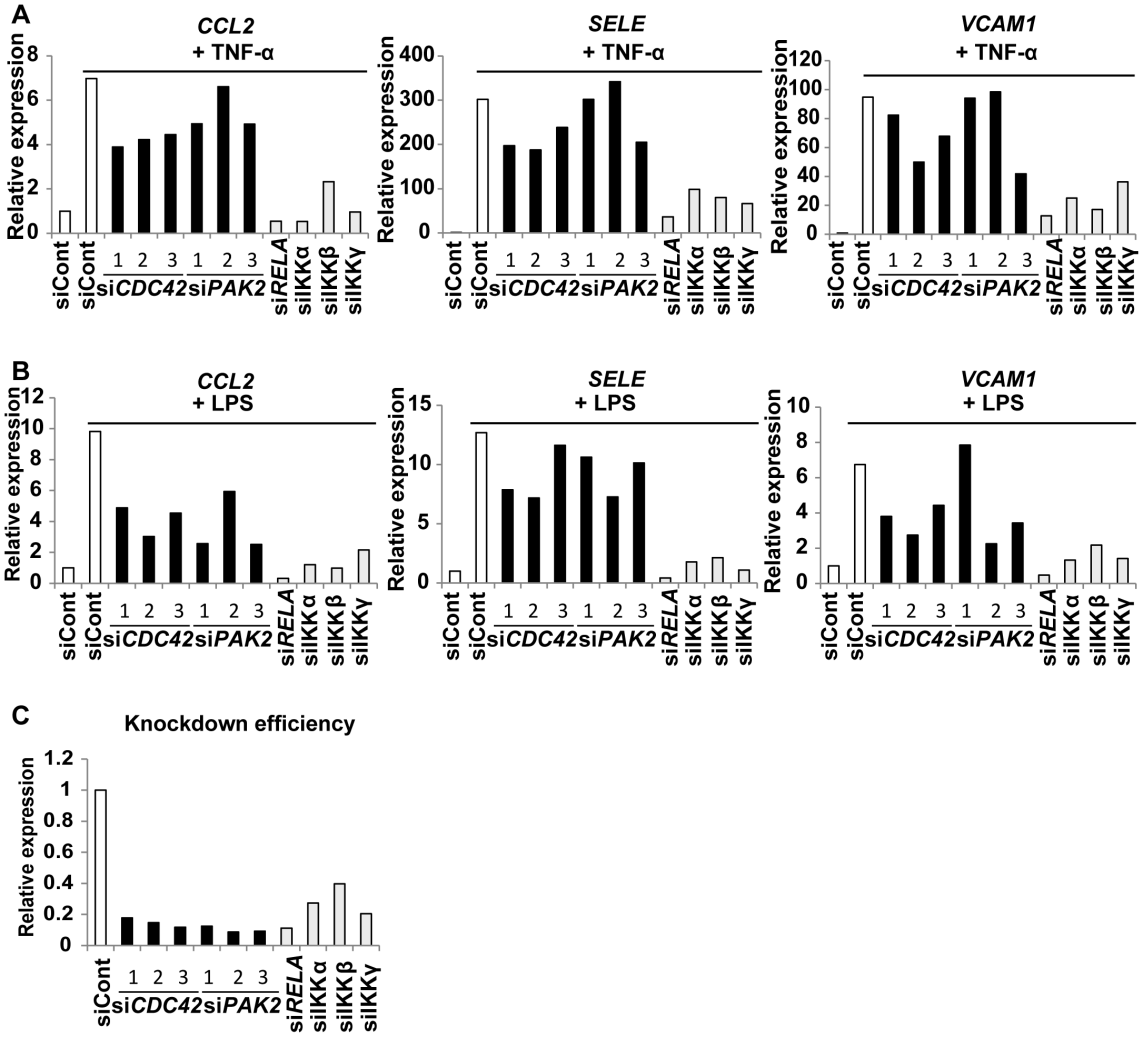
CDC42 signaling has a weaker influence on acute inflammation. (A and B) Human endothelial cells were transduced with siRNA for *CDC42*, *PAK2*, *RELA*, IKKs, or control siRNA (siCont). Cells were treated with TNF-α (A) or LPS (B) at 60 hours after siRNA transduction and were harvested 24 hours later to examine the expression of pro-inflammatory genes by real-time PCR. (C) Knockdown efficiency of siRNAs for *CDC42*, *PAK2*, and NFκB signaling in endothelial cells at 60 hours after transduction. The graph shows expression of each gene in siRNA-treated cells relative to that in siCont-treated cells. Each experiment in Figure 3A–C was repeated three times, and the results were consistent. Representative results are shown.

It is generally accepted that NF-κB is a crucial regulator of acute inflammation induced by infections [23], [24]. To test whether the CDC42 pathway had a role in acute inflammation, we transfected normal endothelial cells with siRNA targeting *CDC42* before treating the cells with TNF-α or LPS (Figure 3A and 3B). In agreement with previous reports [23], [24], knockdown of the NF-κB pathway markedly inhibited the up-regulation of pro-inflammatory molecules in response to stimulation with TNF-α or LPS (Figure 3A and B). In contrast, knockdown of *CDC42* or *PAK2* had a much weaker influence on the response to TNF-α (Figure 3A). Although the response to LPS was modestly attenuated in cells by CDC42 siRNA transfection, we still observed considerable production of inflammatory molecules, especially *SELE* (Figure 3B). Knockdown of siRNAs for *CDC42* or *PAK2* was no less efficient than that of NFκB (Figure 3C), which led us to assume that the differences in intervention for acute inflammatory induction was not merely due to the knockdown efficiency. These results suggest that CDC42 signaling in endothelial cells contributes more specifically to senescence-associated inflammation.

### CDC42 promotes senescence-associated inflammation in mice

**Figure 4:**
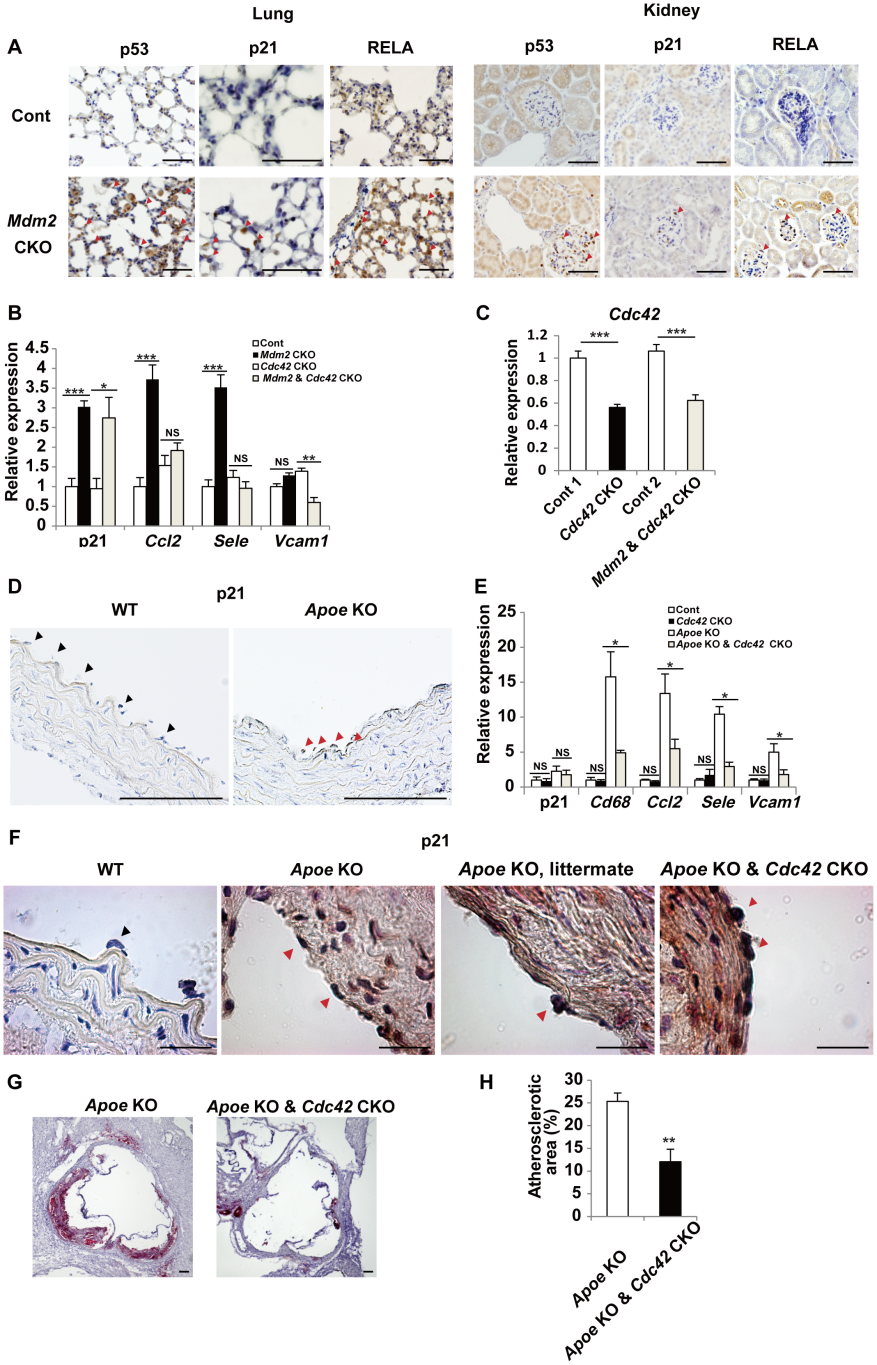
CDC42 signaling regulates chronic inflammation associated with senescence. (A) Immunostaining for p53, p21, and RELA in sections of the lungs and the renal glomeruli from endothelial cell-specific *Mdm2* conditional knockout mice (*Mdm2* CKO, *Pdgfb*-Cre-ER; *Mdm2* ^loxP/loxP^) and their littermate controls (Cont, *Mdm2* ^loxP/loxP^). Arrowheads indicate positive staining of capillary endothelial cells. Scale bar = 100 µm. (B) Expression of p21 and pro-inflammatory genes in the lungs of *Mdm2* CKO mice (*Pdgfb*-Cre-ER; *Mdm2* ^loxP/loxP^), their littermate controls (Cont, *Mdm2* ^loxP/loxP^), *Cdc42* CKO mice (*Pdgfb*-Cre-ER; *Cdc42* ^loxP/loxP^), and *Mdm2* & *Cdc42* CKO mice (*Pdgfb*-Cre-ER; *Mdm2* ^loxP/loxP^; *Cdc42* ^loxP/loxP^) was examined by real-time PCR. n = 4–6. (C) Expression of *Cdc42* was examined by real-time PCR in the lungs of *Cdc42* CKO mice (*Pdgfb*-Cre-ER; *Cdc42* ^loxP/loxP^), their littermate controls (Cont 1, *Cdc42* ^loxP/loxP^), *Mdm2* & *Cdc42* CKO mice (*Pdgfb*-Cre-ER; *Mdm2* ^loxP/loxP^; *Cdc42* ^loxP/loxP^), and their littermate controls (Cont 2, *Mdm2* ^loxP/loxP^; *Cdc42* ^loxP/loxP^). n = 5–6. (D) Immunostaining for p21 in paraffin-embedded sections of the aorta from *Apoe* knockout mice (*Apoe* KO, *Apoe* ^−/−^) and wild-type littermates (WT, *Apoe* ^+/+^). Black arrowheads indicate negative staining of aortic endothelial cells. Red arrowheads indicate positive staining. Scale bar = 100 µm. (E) Expression of *Cdkn1a* (p21), *Cd68,* and pro-inflammatory genes in the aortas of *Cdc42* CKO mice (*Pdgfb*-Cre-ER; *Cdc42* ^loxP/loxP^), their littermate controls (Cont, *Cdc42* ^loxP/loxP^), *Apoe* KO mice (*Apoe* ^−/−^;*Cdc42* ^loxP/loxP^), and *Apoe* KO & *Cdc42* CKO mice (*Apoe* ^−/−^; *Pdgfb*-Cre-ER; *Cdc42* ^loxP/loxP^) was examined by real-time PCR. n = 4–6. (F) Immunostaining for p21 in frozen sections of the aorta from *Apoe* KO mice (*Apoe* ^−/−^), wild-type littermates (WT, *Apoe* ^+/+^), *Apoe* KO littermates (*Apoe* ^−/−^;*Cdc42* ^loxP/loxP^), and *Apoe* KO & *Cdc42* CKO mice (*Apoe* ^−/−^; *Pdgfb*-Cre-ER; *Cdc42* ^loxP/loxP^). Black arrowheads indicate negative staining of aortic endothelial cells for p21. Red arrowheads indicate positive staining for p21. Scale bar = 20 µm. (G) Oil red O staining of aortic sinus sections from *Apoe* KO mice (*Apoe* ^−/−^;*Cdc42* ^loxP/loxP^) and *Apoe* KO & *Cdc42* CKO mice (*Apoe* ^−/−^; *Pdgfb*-Cre-ER; *Cdc42* ^loxP/loxP^). Scale bar = 100 µm. (H) Quantification of the atherosclerotic lesion area relative to the total area at the level of the aortic sinus in *Apoe* KO mice and *Apoe* KO & *Cdc42* CKO mice. n = 5. Data are shown as the mean ± SEM. *P<0.05, **P<0.01, ***P<0.001.

We next examined whether targeting *Cdc42* could inhibit senescence-associated inflammation *in vivo*. We established endothelium-specific *Mdm2* conditional knockout (CKO) mice (*Pdgfb*-Cre-ER; *Mdm2* ^loxP/loxP^). These mutant mice received intraperitoneal injections of tamoxifen once a day for five days from 6–8 weeks of age to induce Cre-mediated recombination and were analyzed after 3 weeks. Endothelial expression of Mdm2 (a negative regulator of p53 expression [25]) was abolished by treatment with tamoxifen, leading to up-regulation of the endothelial expression of p53 and p21, which are key indicators of cellular senescence. Nuclear staining for p53 and p21 was strong in the endothelial cells of most blood vessels in capillary-rich organs such as the lungs and the glomeruli of the kidneys (Figure 4A), while the endothelium of vessels in the liver was not stained for either p53 or p21 (data not shown), consistent with a previous report [26]. Immunohistochemistry also revealed an increase in the nuclear translocation of NF-κB (RelA) in *Mdm2* CKO mice (Figure 4A). We measured the expression of mRNA for p21 and pro-inflammatory genes in the lungs of *Mdm2* CKO mice after 3 weeks of tamoxifen treatment by real-time PCR. Consistent with the findings in senescent human endothelial cells, there was a marked increase of *CCL2* and *SELE* associated with up-regulation of p21 (Figure 4B). To further investigate the role of *Cdc42* in *Mdm2* CKO mice (*Pdgfb*-Cre-ER; *Mdm2* ^loxP/loxP^), we crossed floxed *Cdc42* mice (*Cdc42* ^loxP/loxP^) to obtain *Mdm2 Cdc42* CKO mice (*Pdgfb*-Cre-ER; *Mdm2* ^loxP/loxP^; *Cdc42* ^loxP/loxP^) and *Cdc42* CKO mice (*Pdgfb*-Cre-ER; *Cdc42* ^loxP/loxP^). As a result, we confirmed a decrease of *Cdc42* expression in the lungs of both *Cdc42* CKO mice and *Mdm2 Cdc42* CKO mice (Figure 4C). The inflammatory responses observed in *Mdm2* CKO mice was significantly attenuated in *Mdm2 Cdc42* CKO mice, whereas up-regulation of p21 was not affected by deletion of *Cdc42* (Figure 4B), suggesting that Cdc42 deletion inhibits p53-induced up-regulation of pro-inflammatory gene expression without having any influence on cell cycle regulation in this mouse model. *VCAM1* was not up-regulated in *Mdm2* CKO mice, unlike in senescent human endothelial cells, but was significantly decreased in double CKO mice compared with their littermates (Figure 4B). These results suggested that Cdc42 mediates p53-induced vascular inflammation in vivo.

To test whether endothelial cell Cdc42 deletion also inhibited chronic inflammation in a mouse model of atherosclerosis, we used apolipoprotein E knockout (*Apoe* KO) mice in which atherosclerotic plaque formation was enhanced by endothelial NF-κB signaling [27]. Accumulation of p21 proteins and senescence-associated β-galactosidase positive cells has also been reported in the aorta of this model [28]. Histological examination showed that nuclear expression of p21 was increased in the aortic endothelial cells of *Apoe* KO mice compared with wild-type mice (WT) (Figure 4D), supporting the notion that pro-senescence signaling is enhanced in the endothelium of atherosclerotic plaque. To investigate the role of Cdc42 in *Apoe* KO mice, we crossed endothelial cell-specific *Cdc42* CKO mice (*Pdgfb*-Cre-ER; *Cdc42* ^loxP/loxP^) with *Apoe* KO mice (*Apoe* ^−/−^) to establish Apoe KO Cdc42 CKO mice (*Apoe* ^−/−^; *Pdgfb*-Cre-ER; *Cdc42* ^loxP/loxP^). We then fed *Cdc42* CKO mice, their littermate controls (Cont), *Apoe* KO mice, and *Apoe* KO *Cdc42* CKO mice a high-cholesterol diet from 6–8 weeks of age and we simultaneously deleted endothelial *Cdc42* by treatment with tamoxifen. The aortic tissues were harvested for analysis of RNA and histological examination after 8 weeks (at 14–16 weeks old). To investigate the influence of *Cdc42* deletion on inflammation, we measured the expression of the macrophage surface marker *Cd68* by real-time PCR. The results showed that *Cd68* expression was markedly increased in *Apoe* KO mice, while this increase was significantly attenuated in *Apoe* KO *Cdc42* CKO mice (Figure 4E), suggesting that infiltration of macrophages into the aorta was significantly reduced by deletion of *Cdc42*. Up-regulation of pro-inflammatory genes in the aorta was also attenuated in *Apoe* KO *Cdc42* CKO mice compared with *Apoe* KO mice (Figure 4E). In contrast, expression of pro-inflammatory molecules did not differ between *Cdc42* CKO mice and their littermate controls (Figure 4E), suggesting that Cdc42 signaling was specifically activated by atherogenic stimuli, thereby provoking chronic inflammation. Aortic expression of p21 mRNA in *Apoe* KO mice was not altered by deletion of *Cdc42* (Figure 4E). Likewise, histological examination demonstrated that enhanced nuclear staining for p21 in the endothelial cells of *Apoe* KO mice was not attenuated by endothelial deletion of *Cdc42* (Figure 4F), indicating that ablation of Cdc42 did not affect cell cycle arrest. This conclusion was strengthened by the results of immunostaining for γ-H2AX, a marker of DNA damage and cellular senescence, since the expression of γ-H2AX was increased in the endothelium of *Apoe* KO mice and this increase was not affected by deletion of *Cdc42* (Figure S3). Thus, it is likely that atherogenic stimuli promote endothelial senescence and that Cdc42 is a mediator of chronic inflammation associated with endothelial senescence. We then further analyzed the influence of Cdc42 on the development of atherosclerosis. When cross-sections of the aortic sinus were stained with Oil red O to visualize atherosclerotic plaques, we found that the mean atherosclerotic lesion area was significantly smaller in *Apoe* KO *Cdc42* CKO mice than in their littermate controls (*Apoe* KO) (Figure 4G and H). These results suggested a crucial role of endothelial Cdc42 in chronic inflammation and the progression of atherosclerosis.

### CDC42 promotes senescence-associated inflammation in worms

**Figure 5:**
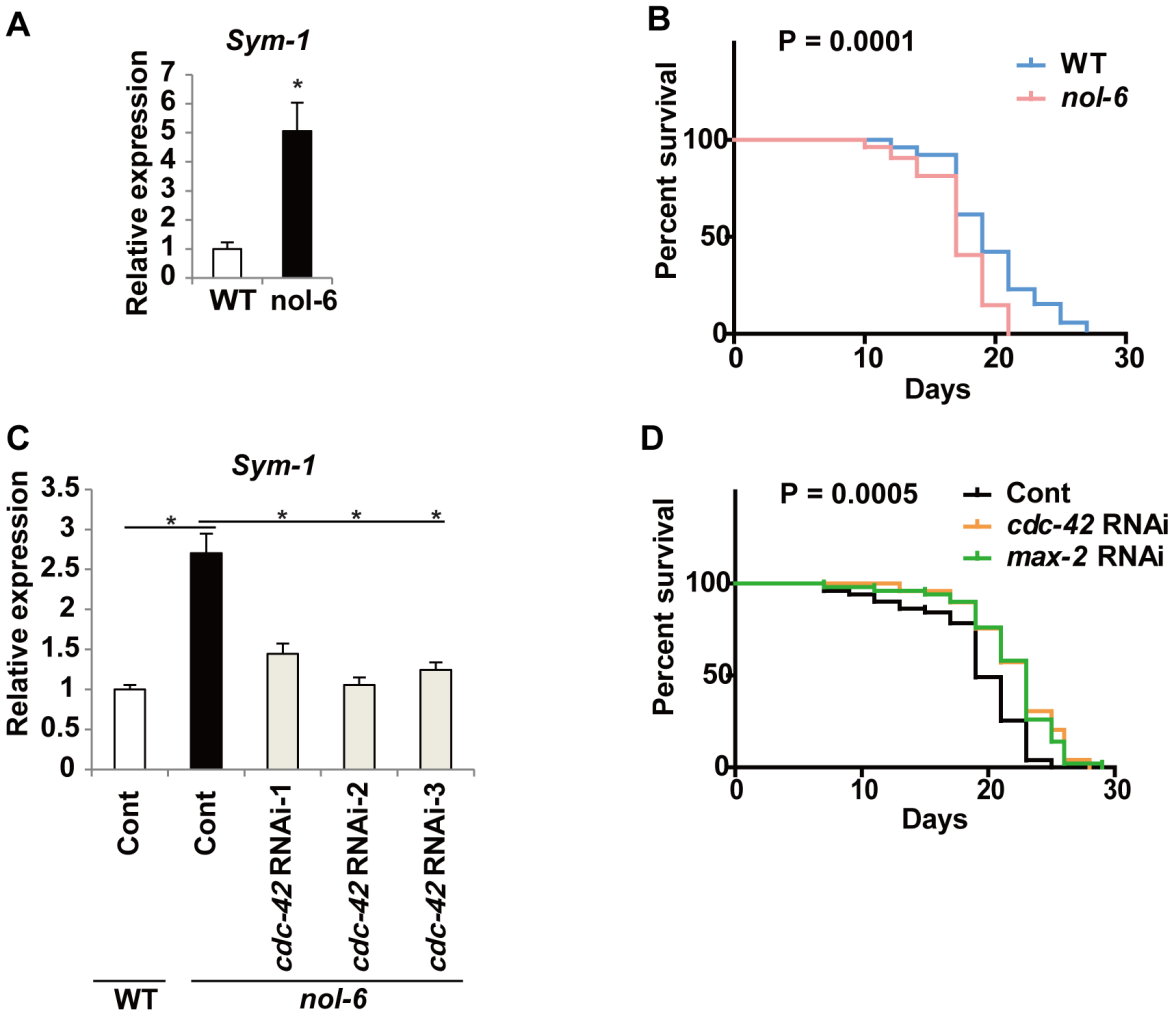
CDC42 promotes senescence-associated inflammation in worms. (A) Expression of *sym-1* in wild-type (WT) and *nol-6* mutant worms was examined by real-time PCR on day 9. n = 3. (B) Survival curves of wild-type (WT) and *nol-6* mutant worms maintained at 22°C. n = 50. (C) Expression of *sym-1* by wild-type (WT) and *nol-6* mutants treated with RNAi for *cdc-42* or control RNAi (Cont) was examined by real-time PCR. mRNA was analyzed 48 hours after starting feeding RNAi. n = 4. (D) Survival curves of *nol-6* mutant worms treated with RNAi for *cdc-42*, *max-2*, or control RNAi (Cont). n = 50. Data are shown as the mean ± SEM. *P<0.05, **P<0.01, ***P<0.001.

To further investigate the role of CDC42 in inflammation associated with senescence, we examined the influence of CDC42 deletion on aging and inflammation. The nematode *C. elegans* has recently been recognized as an excellent model for studying longevity and aging-associated phenotypes, mainly because of its short lifespan, availability of various genetic mutants, and amenability to genetic manipulation by RNA-interference (RNAi). We utilized *nol-6* mutant worms as a potential model of chronic inflammation that could mimic the phenotypic features of senescent human endothelial cells and *Apoe* KO mice, because this mutant displays over-activation of innate immunity (the counterpart of inflammation) via a p53/cep-1-dependent pathway [29]. In this mutant, there was increased expression of inflammatory molecules such as *sym-1* (Figure 5A), as described previously [29], which is crucial for p53-dependent activation of innate immunity [29]. The lifespan of *nol-6* mutants raised at 22°C was significantly shorter than that of wild-type worms (Figure 5B), presumably due to the adverse influence of enhanced inflammation [29]. Knockdown of *cdc-42* or *max-2* (a homolog of *PAK2*) by feeding RNAi from the adult stage significantly down-regulated *sym-1* expression (Figure 5C and data not shown) and extended the lifespan of *nol-6* mutant worms to that of normal worms (Figure 5D and data not shown), further supporting a role of *cdc-42* in senescence-associated inflammation *in vivo*.

## Discussion

The present findings provide evidence that the CDC42 pathway is critically involved in chronic inflammation induced by cellular aging signals. Inhibition of this pathway significantly attenuated the sustained up-regulation of inflammatory molecules in senescent human endothelial cells, as well as in murine models of cellular senescence and atherosclerosis, and in short-lived mutant worms. Thus, this inflammatory program appears to be conserved among species.

Despite evidence that accumulation of senescent endothelial cells occurs in atherosclerotic lesions and that senescent endothelial cells display the pro-inflammatory phenotype relevant to atherosclerosis [5], [30], it has been unclear how senescence of endothelial cells influences the development of atherosclerosis. The present study identified CDC42 as a non-canonical pathway that activates NF-κB and up-regulates pro-inflammatory genes in senescent endothelial cells. We found that p53-induced vascular inflammation was significantly ameliorated by deletion of Cdc42 signaling. We also demonstrated that inhibition of Cdc42 signaling in the endothelium decreased vascular inflammation and plaque formation in a mouse model of atherosclerosis. It is interesting that deletion of CDC42 signaling had a much weaker influence on acute inflammation than chronic inflammation. Although the alleviated inflammation by Cdc42 deletion might also involve a declined response to exogenous ligands such as oxidized LDL [31], this work supports the notion that the Cdc42-dependet pro-inflammatory pathway is specifically activated by senescence-associated stimuli such as the risk factors for atherosclerosis.

Morphological change is one of the cardinal features of the senescent phenotype. It was reported that CDC42 was activated in senescent cells, contributing to morphological change [32]. Recently, CDC42 activation has also been implicated in the features of aging in mice and humans [20], [21]. An age-associated increase of CDC42 contributes to the decline of hematopoietic stem cell function with aging [19]. It has been reported that disruption of Cdc42 GTPase-activating protein (Cdc42GAP), a negative regulator of Cdc42, leads to constitutive activation of CDC42 in various tissues of mice, as well as a premature aging-like phenotype and shortened lifespan [21]. In contrast to our *in vitro* experiments of endothelial cells, disruption of Cdc42GAP in mouse embryonic fibroblasts provoked premature senescence by activating p53 [21]. While CDC42 activation of NF-κB was also previously noted [33], [34], CDC42 does not necessarily mediate NF-κB activation [33], [35]. Thus, the role of CDC42 in inflammation and cellular senescence would be both cell type and context dependent.

Elimination of pre-senescent and senescent cells delays the onset of various age-related pathological conditions [12]. Our results suggested that inhibition of specific signaling in senescent cells is sufficient to ameliorate age-related diseases such as atherosclerosis. Because deletion of CDC42 had no effect on cell proliferation and only a weak influence on acute inflammation, CDC42 could be an attractive target for the treatment of age-associated diseases without promoting tumor formation or compromising normal immune function.

## Methods

### Cell culture, viral infection, and siRNA transfection

Human umbilical vein endothelial cells (HUVEC, Eidia) were maintained in EBM-2 (Lonza) supplemented with EGM-2 SingleQuots (Lonza) in dishes coated with gelatin (Sigma). In some experiments, LPS (1 µg/ml, Sigma) or TNF-α (2 ng/ml, eBioscience) was added to the culture medium and the cells were harvested for real-time PCR after 24 hours. Retroviral and adenoviral transduction was performed as described previously [36], [37]. Briefly, cyclin-dependent kinase inhibitor 2A (p16) was cloned into the pBabe-puro retroviral vector and cyclin-dependent kinase inhibitor 1A (p21) and a constitutively active form of CDC42 (CDC42 V12) were cloned into the pLNCX vector. The respective empty vectors were used as controls. Retroviral stock solutions were supplemented with 8 µg/ml polybrene (Sigma, Tokyo, Japan) and cultured with HUVEC for 24 hours. Then infected cells were selected by culture for four days with 0.8 µg/ml puromycin for the pBabe-puro-based retroviral vector and with 500 µg/ml G418 for the pLNCN-based vectors. At six days after retroviral infection, siRNAs purchased from Ambion or Invitrogen were transfected at 10 nmol/L with RNAiFect (Qiagen) or Lipofectamine RNAiMAX (Invitrogen) according to the manufacturers’ instructions. Cells were analyzed at 72 hours after transfection. The sequences of the siRNAs targeting CDC42 were UGGUGCUGUUGGUAAAACA, UGAGAUAACUCACCACUGU, and CAGUUAUGAUUGGUGGAGA; the sequences for PAK2 were GAACUGAUCAUUAACGAGA, GGUGAUGAAAGAAUUGAAA, and CAGAGGUGGUUACACGGAA; and the sequences for NF-κB signaling were CCCUUUACGUCAUCCCUGA, GGAGUACCCUGAGGCUAUA, GCCCUAUCCCUUUACGUCA (RELA), GGACUAAAAGAAGACUAUA (IKK-α), GACUUGAAUGGAACGGUGA (IKK-β), and GAUUGUGAUGGAGACCGUU (IKK-γ). High titer adenoviral stocks were generated with the Adeno-X Expression System (Clontech) according to the manufacturer’s instructions. Infection was done for one hour with adenoviral stock solutions containing the dominant-negative form of CDC42, which were then replaced with normal culture medium.

### Western blot analysis

Whole cell lysates were resolved by SDS polyacrylamide gel electrophoresis (PAGE). Proteins were transferred onto polyvinylidene difluoride (PVDF) membranes (Millipore) and were incubated with the primary antibody, followed by incubation with the specific horseradish peroxidase-conjugated immunoglobulin G antibody (anti-mouse, anti-rabbit, or anti- goat; Jackson). Specific proteins were detected by enhanced chemiluminescence (Amersham). Antibodies used for western blotting were as follows: anti-RELA antibody (Cell Signaling), anti-phospho-Ser536 RELA antibody (Cell Signaling), anti-PAK2 antibody (Cell Signaling), anti-phospho-ser20 PAK2 antibody (Cell Signaling), anti-CDC42 antibody (Cell Signaling), anti-histone H3 (Cell Signaling), and anti-GAPDH antibody (Santa Cruz). Nuclear and cytoplasmic extracts were prepared by using NE-PER nuclear and cytoplasmic extraction reagents (Pierce) to detect nuclear translocation of RELA. GTP-bound CDC42 was measured with the Active Cdc42 Pull-Down and Detection Kit (Thermo-Scientific). Briefly, whole cell lysates were prepared from human endothelial cells at 6 days after infection with pLNCX-p21 or pLNCX (Mock). The lysates were incubated with the GST-Pak1 binding domain fusion protein and glutathione resin to enable isolation of the target active (GTP-bound) GTPase. Unbound lysate proteins, including inactive or GDP-bound GTPase, were removed by using the spin columns and active GTPase was recovered from the glutathione resin by using SDS-PAGE loading buffer and analyzed by Western blotting. Then the immunoblot bands were quantified with Image J software.

### Animal models

The animal study protocols were approved by the Chiba University Review Board and by the Committee on the Ethics of Animal Experiments of Chiba University (Permits Number: 21–254, 22–213, 23–88, 24–174, and 25–217). All mice were housed and maintained under pathogen-free conditions. Floxed *Cdc42* mice (*Cdc42* ^loxP/loxP^), floxed *Mdm2* mice (*Mdm2* ^loxP/loxP^), and *Pdgfb*-Cre-ER mice were generated as described previously [26], [38], [39]. *Apoe* knockout mice were obtained from the Jackson Laboratory. The genetic background of floxed *Cdc42* mice was a hybrid of C57BL/6, ICR, and 129/Ola, while that of floxed *Mdm2* mice was a hybrid of C57BL/6, FVB, and 129S7/SvEvBrd, that of *Pdgfb*-Cre-ER mice was a hybrid of CBA and C57BL/6, and that of *Apoe* knockout mice was C57BL/6. Floxed *Mdm2* mice and *Pdgfb*-Cre-ER mice were backcrossed to C57BL/6 four times after being transferred to Chiba University. *Pdgfb*-Cre-ER mice were crossed with mice carrying floxed alleles to generate endothelial cell-specific gene knockout mice. In this model, the efficiency of tamoxifen-induced Cre recombinase activity has previously been tested with ROSA26-lacZ reporter mice, revealing that recombination was achieved in most of the endothelial cells of the capillaries and small arterioles in adult animals [26], [40]. Although endogenous Pdgfb is also expressed by non-endothelial cells, it was previously demonstrated that transgene expression is endothelial cell-specific in various tissues including skeletal muscle, except for the liver [26]. We established endothelium-specific *Mdm2* conditional knockout (CKO) mice (*Pdgfb*-Cre-ER; *Mdm2* ^loxP/loxP^). To study the role of *Cdc42* in *Mdm2* CKO mice (*Pdgfb*-Cre-ER; *Mdm2* ^loxP/loxP^), we further crossed Cdc42 floxed mice (*Cdc42* ^loxP/loxP^) to obtain *Mdm2 Cdc42* CKO mice (*Pdgfb*-Cre-ER; *Mdm2* ^loxP/loxP^; *Cdc42* ^loxP/loxP^) and *Cdc42* CKO mice (*Pdgfb*-Cre-ER; *Cdc42* ^loxP/loxP^). These mutant mice received 4 mg of 4-hydroxytamoxifen (Sigma) intraperitoneally once a day for five days from 6–8 weeks of age to induce Cre-mediated recombination and were analyzed 3 weeks after treatment. Each experimental group was compared with their littermate controls. Before harvesting of tissue samples, mice were anesthetized with urethane (1 g/kg) and perfused with PBS. To study the role of *Cdc42* in *Apoe* knockout mice, we established *Apoe* KO *Cdc42* CKO mice (*Apoe* ^−/−^; *Pdgfb*-Cre-ER; *Cdc42* ^loxP/loxP^). *Apoe* KO mice (*Apoe* ^−/−^), their wild-type littermates (WT, *Apoe* ^+/+^), *Cdc42* CKO mice (*Pdgfb*-Cre-ER; *Cdc42* ^loxP/loxP^), their littermate controls (Cont, *Cdc42* ^loxP/loxP^), *Apoe* KO littermate mice (*Apoe* ^−/−^; *Cdc42* ^loxP/loxP^), and *Apoe* KO *Cdc42* CKO mice (*Apoe* ^−/−^; *Pdgfb*-Cre-ER; *Cdc42* ^loxP/loxP^) were fed a high-fat diet containing 1.25% cholesterol and 0.5% cholate (Oriental Kobo) from 6 to 8 weeks of age and then maintained for 8 weeks before analysis. Mice were killed by cervical dislocation and perfused with PBS. The aortas were dissected and cleaned of adherent connective tissue under a dissecting microscope. Then the proximal half of each aorta was homogenized for analysis of the expression of target markers.

### Histological analysis

For immunohistochemistry, sections of paraffin-embedded or frozen tissues were incubated with antibodies for p53 (Vector Laboratories), p21 (BD Pharmingen and Abcam), RelA (Santa Cruz), and anti-γH2AX (Cell Signaling) after antigen retrieval by heating in citrate buffer. For analysis of atherosclerotic plaques in *Apoe* KO mice, 7 frozen cross-sections of the aortic sinus (10 µm thick) from each mouse were stained with Oil Red O (Sigma). Then the average lesion area was quantified with Image J software.

### Worms

Bristol N2 wild-type and *nol-6* (AY1) strains were provided by Dr. Ishii and the Caenorhabditis Genetic Center, respectively. Temperature-sensitive *nol-6* mutants were maintained at 15°C. Lifespan experiments were performed at 22°C with 50 animals per condition. Synchronized L1 worms were fed with OP50. After the worms reached the young adult stage, FUdR (0.5 mg/ml) was added to the plate to prevent the production of progeny. On day 6, adult worms were placed on NGM plates containing IPTG (1 mmol/L) and carbenicillin (25 µg/ml) seeded with HT115 (DE3) bacteria carrying feeding RNAi plasmids or control L4440 vectors (Thermo-Scientific and DNAFORM). Survival was assessed every second or third day.

### Real-time PCR

RNA was extracted from human cells, mouse tissues, or worms using RNABee (Tel-Test) and was transcribed to cDNA using a QuantiTect reverse transcription kit (Qiagen). Quantitative real-time PCR was performed with the Universal ProbeLibrary and a LightCycler 480 (Roche Applied Science). GAPDH was used to normalize the RNA content of human and mouse samples, while H20J04.3 [41] was applied for worm samples.

### Statistical analysis

Data are shown as the mean ± SEM. The two-tailed Student’s *t*-test or one way ANOVA was used to assess statistical significance (*P<0.05, **P<0.01, or ***P<0.001). For lifespan analysis of worms, the significance of differences was assessed by the log-rank (Mantel–Cox) test.

## Supporting Information

Figure S1 **Expression of cyclin-dependent kinases and knockdown efficacy in senescent human endothelial cells.** (A) Human endothelial cells were infected with an empty vector (Mock) or a retroviral vector encoding cyclin-dependent kinase inhibitor 1A (p21) or cyclin-dependent kinase inhibitor 2A (p16) to induce senescence. Expression of p21 and p16 was examined by real-time PCR at 6 days after infection. Data are shown as the mean ± SEM. n = 5. (B) Human endothelial cells were infected with a retroviral vector encoding p21 to induce senescence. Six days after infection, the cells were transduced with 3 sets of siRNAs for *RELA* (1–3), siRNAs for IKKs (α, β, γ subunits), siRNAs for *Cdc42* (1–3), siRNAs for *PAK2* (1–3), or control siRNA (siCont). Expression of target genes was examined by real-time PCR after 72 hours. The graph shows expression of each gene in siRNA-treated cells relative to that in siCont-treated cells. Data are shown as the mean ± SEM. n = 3. (C) Human endothelial cells were infected with a retroviral vector encoding p21 to induce senescence. Six days after infection, the cells were transduced with 6 sets of siRNAs for *ATM* (1–6), siRNAs for *CHEK2* (1–6), or control siRNA (siCont). Expression of target genes was examined by real-time PCR after 72 hours. The graph shows expression of each gene in siRNA-treated cells relative to that in siCont-treated cells.
(DOCX)

Figure S2 **Knockdown efficacy in human endothelial cells infected with active CDC42.** Human endothelial cells were infected with a retroviral vector encoding active CDC42 (CDC42 V12). Six days after infection, the cells were transduced with siRNAs for *RELA*, IKKs (α, β, γ subunits), or control siRNA (siCont). Expression of the target genes was examined by real-time PCR after 72 hours. The graph shows expression of each gene in siRNA-treated cells relative to that in siCont-treated cells. Data are shown as the mean ± SEM. n = 3.
(DOCX)

Figure S3 **Expression of γ-H2AX in the aorta.** Immunostaining for γ-H2AX in sections of the aorta from *Apoe* KO mice (*Apoe* ^−/−^), wild-type littermates (WT, *Apoe* ^+/+^), *Apoe* KO littermates (*Apoe* ^−/−^;*Cdc42* ^loxP/loxP^), and *Apoe* KO & *Cdc42* CKO mice (*Apoe* ^−/−^; *Pdgfb*-Cre-ER; *Cdc42* ^loxP/loxP^). Black arrowheads indicate negative staining of aortic endothelial cells for p21. Red arrowheads indicate positive staining for p21. Scale bar = 20 µm.
(DOCX)

Table S1 **The gene list of siRNA screening.**
(PDF)
