# Supplementary material for: A Crucial Role for CDC42 in Senescence-Associated Inflammation and Atherosclerosis
Source: PLoS One. 2014 Jul 24;9(7):e102186. doi: 10.1371/journal.pone.0102186 (PMC4109913; doi:10.1371/journal.pone.0102186)
Supplement: Figure S1 — Expression of cyclin-dependent kinases and knockdown efficacy in senescent human endothelial cells. (A) Human endothelial cells were infected with an empty vector (Mock) or a retroviral vector encoding cyclin-dependent kinase inhibitor 1A (p21) or cyclin-dependent kinase inhibitor 2A (p16) to induce senescence. Expression of p21 and p16 was examined by real-time PCR at 6 days after infection. Data are shown as the mean ± SEM. n = 5. (B) Human endothelial cells were infected with a retroviral vector encoding p21 to induce senescence. Six days after infection, the cells were transduced with 3 sets of siRNAs for RELA (1–3), siRNAs for IKKs (α, β, γ subunits), siRNAs for Cdc42 (1–3), siRNAs for PAK2 (1–3), or control siRNA (siCont). Expression of target genes was examined by real-time PCR after 72 hours. The graph shows expression of each gene in siRNA-treated cells relative to that in siCont-treated cells. Data are shown as the mean ± SEM. n = 3. (C) Human endothelial cells were infected with a retroviral vector encoding p21 to induce senescence. Six days after infection, the cells were transduced with 6 sets of siRNAs for ATM (1–6), siRNAs for CHEK2 (1–6), or control siRNA (siCont). Expression of target genes was examined by real-time PCR after 72 hours. The graph shows expression of each gene in siRNA-treated cells relative to that in siCont-treated cells. (DOCX) [file pone.0102186.s001.docx]

**
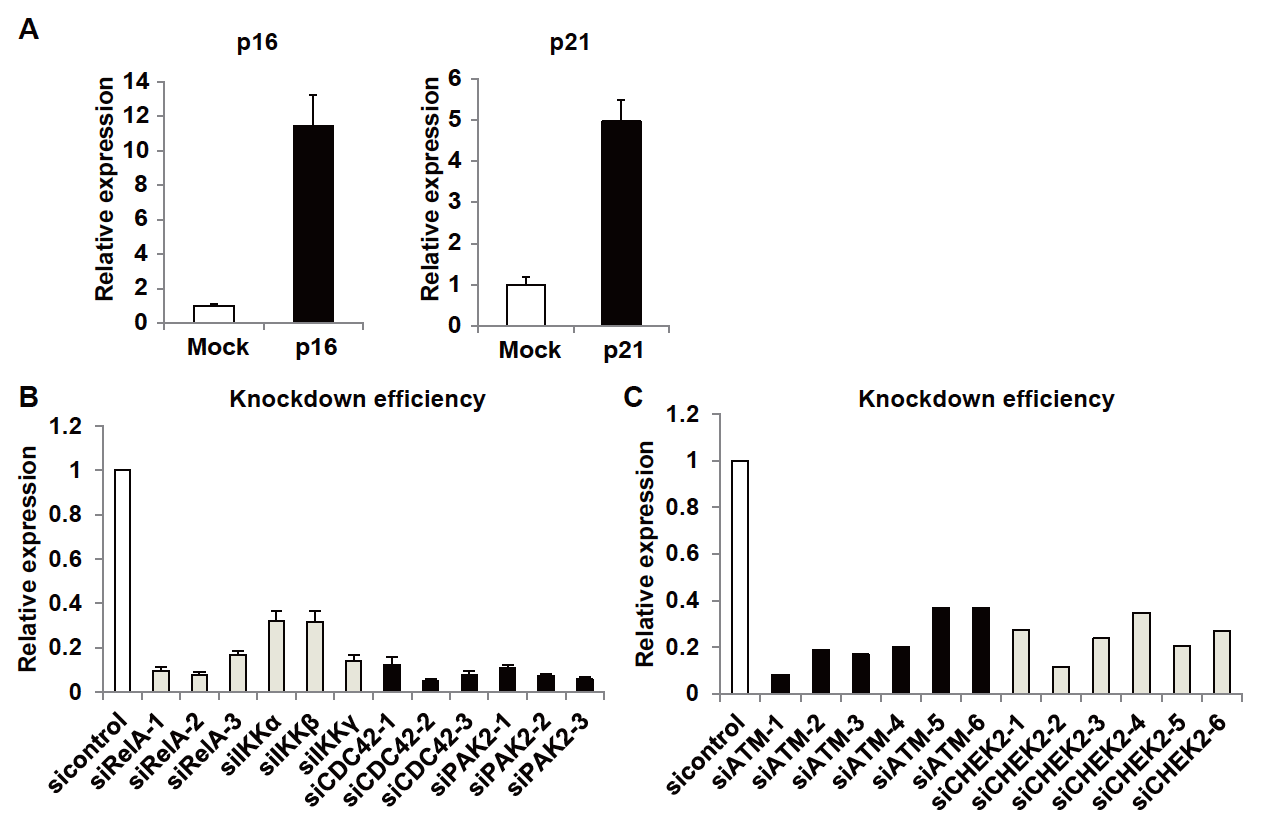
**

**Figure S1. Expression of cyclin-dependent kinases and knockdown efficacy in senescent human endothelial cells.**

(A) Human endothelial cells were infected with an empty vector (Mock) or a retroviral vector encoding cyclin-dependent kinase inhibitor 1A (p21) or cyclin-dependent kinase inhibitor 2A (p16) to induce senescence. Expression of p21 and p16 was examined by real-time PCR at 6 days after infection. Data are shown as the mean ± SEM. n=5. (B) Human endothelial cells were infected with a retroviral vector encoding p21 to induce senescence. Six days after infection, the cells were transduced with 3 sets of siRNAs for *RELA* (1–3), siRNAs for IKKs (α, β, γ subunits), siRNAs for *Cdc42* (1–3), siRNAs for *PAK2* (1–3), or control siRNA (siCont). Expression of target genes was examined by real-time PCR after 72 hours. The graph shows expression of each gene in siRNA-treated cells relative to that in siCont-treated cells. Data are shown as the mean ± SEM. n=3. (C) Human endothelial cells were infected with a retroviral vector encoding p21 to induce senescence. Six days after infection, the cells were transduced with 6 sets of siRNAs for *ATM* (1–6), siRNAs for *CHEK2* (1–6), or control siRNA (siCont). Expression of target genes was examined by real-time PCR after 72 hours. The graph shows expression of each gene in siRNA-treated cells relative to that in siCont-treated cells.
