# Supplementary material for: A Crucial Role for CDC42 in Senescence-Associated Inflammation and Atherosclerosis
Source: PLoS One. 2014 Jul 24;9(7):e102186. doi: 10.1371/journal.pone.0102186 (PMC4109913; doi:10.1371/journal.pone.0102186)
Supplement: Figure S2 — Knockdown efficacy in human endothelial cells infected with active CDC42. Human endothelial cells were infected with a retroviral vector encoding active CDC42 (CDC42 V12). Six days after infection, the cells were transduced with siRNAs for RELA, IKKs (α, β, γ subunits), or control siRNA (siCont). Expression of the target genes was examined by real-time PCR after 72 hours. The graph shows expression of each gene in siRNA-treated cells relative to that in siCont-treated cells. Data are shown as the mean ± SEM. n = 3. (DOCX) [file pone.0102186.s002.docx]

**
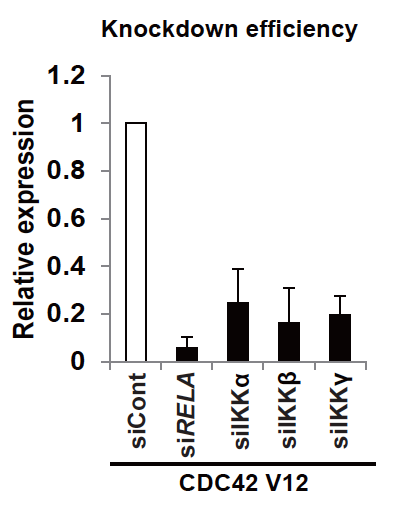
**

**Figure S2. Knockdown efficacy in human endothelial cells infected with active CDC42.**

Human endothelial cells were infected with a retroviral vector encoding active CDC42 (CDC42 V12). Six days after infection, the cells were transduced with siRNAs for *RELA*, IKKs (α, β, γ subunits), or control siRNA (siCont). Expression of the target genes was examined by real-time PCR after 72 hours. The graph shows expression of each gene in siRNA-treated cells relative to that in siCont-treated cells. Data are shown as the mean ± SEM. n=3.
