# Supplementary material for: A Crucial Role for CDC42 in Senescence-Associated Inflammation and Atherosclerosis
Source: PLoS One. 2014 Jul 24;9(7):e102186. doi: 10.1371/journal.pone.0102186 (PMC4109913; doi:10.1371/journal.pone.0102186)
Supplement: Figure S3 — Expression of γ-H2AX in the aorta. Immunostaining for γ-H2AX in sections of the aorta from Apoe KO mice (Apoe −/−), wild-type littermates (WT, Apoe +/+), Apoe KO littermates (Apoe −/−;Cdc42 loxP/loxP), and Apoe KO & Cdc42 CKO mice (Apoe −/−; Pdgfb-Cre-ER; Cdc42 loxP/loxP). Black arrowheads indicate negative staining of aortic endothelial cells for p21. Red arrowheads indicate positive staining for p21. Scale bar = 20 µm. (DOCX) [file pone.0102186.s003.docx]

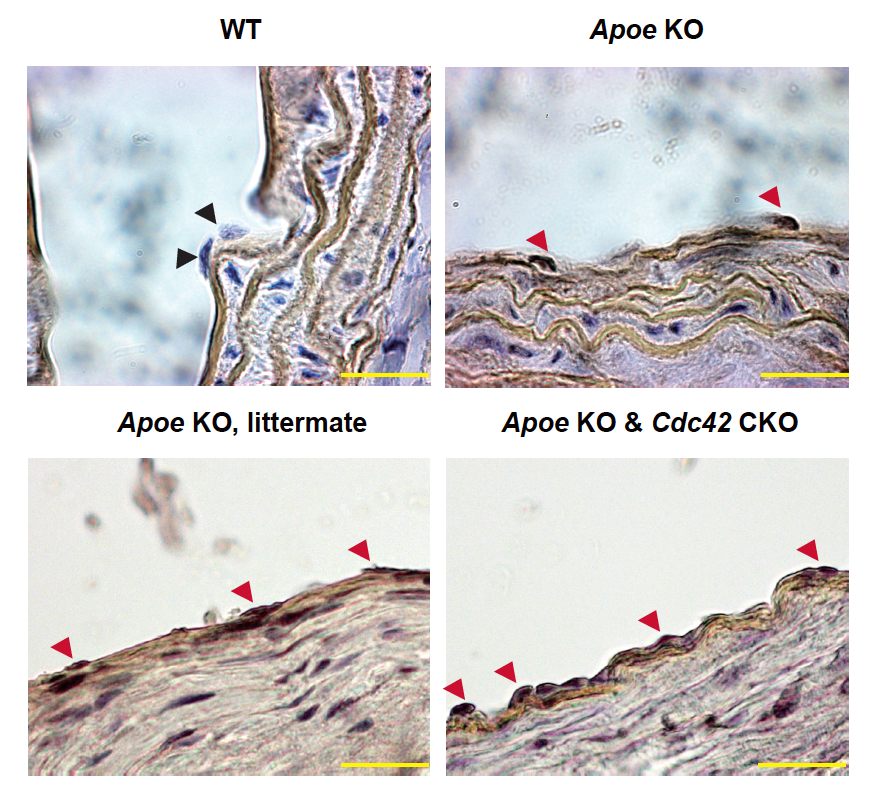


**Figure S3. Expression of γ-H2AX in the aorta.**

Immunostaining for γ-H2AX in sections of the aorta from *Apoe* KO mice (*Apoe*^–/–^), wild-type littermates (WT, *Apoe*^+/+^), *Apoe* KO littermates (*Apoe*^–/–^;*Cdc42*^loxP/loxP^), and *Apoe* KO & *Cdc42* CKO mice (*Apoe*^–/–^; *Pdgfb*-Cre-ER; *Cdc42*^loxP/loxP^). Black arrowheads indicate negative staining of aortic endothelial cells for p21. Red arrowheads indicate positive staining for p21. Scale bar = 20 µm.
