# Supplementary material for: A Crucial Role for CDC42 in Senescence-Associated Inflammation and Atherosclerosis
Source: PLoS One. 2014 Jul 24;9(7):e102186. doi: 10.1371/journal.pone.0102186 (PMC4109913; doi:10.1371/journal.pone.0102186)
Supplement: Table S1 — The gene list of siRNA screening. (PDF) [file pone.0102186.s004.pdf]

Table S1.　　The gene list of RNAi screening.

ADRB2
AGER
ALDOA
ALDOB
ALDOC
ANXA2
ASH1L
ATF2
ATM
ATP5A1
ATR
CALM1
CCL2
CDC42
CDK1
CDK2
CDK3
CDK4
CDK5
CDK6
CDKN1A (p21)
CDKN2A (p16)
CEBPB
CHEK1
CHEK2
CHEK2
CHUK
COX5B
CREBBP
DDX58
DNMT1
E2F1
E2F2
E2F3
E2F4
E2F5
E2F6
E2F7
E2F8
EFNB1
EFNB2
EFNB3

ELK1
EP300
EPHB4
ERC1
ERN1
ETNK1
ETNK2
EZH2
FGF2
FOXO1
FOXO3
FOXO4
GK
GK2
HDAC3
IKBKB
IKBKE
IKBKG
IL8
IL8RB
IRAK1
IRAK4
IRF1
IRF3
IRF5
JAK1
JAK2
JAK3
JUN
JUNB
JUND
KDR
LIFR
LMNA
LYPLA1
MAP2K1
MAP2K2
MAP2K3
MAP2K4
MAP2K5
MAP2K6
MAP2K7
MAP3K1

MAP3K10
MAP3K11
MAP3K12
MAP3K13
MAP3K14
MAP3K2
MAP3K3
MAP3K4
MAP3K5
MAP3K6
MAP3K7
MAP3K7IP2
MAP3K8
MAP3K9
MAP4K1
MAP4K2
MAP4K3
MAP4K4
MAPK10
MAPK11
MAPK14
MAPK7
MAPK8
MAPK9
MEOX1
MLL
MLL4
MYC
MYD88
NDUFA1
NDUFS1
NFATC1
NFKB1
NFKB2
NLRP1
NOD2
PAK1
PAK2
PAK3
PAK4
PFKL
PFKM
PFKP

PGK1
PGK2
PHLDA1
PRKCD
PRKDC
PTEN
PTK2
PXN
RAC1
RB1
RELA
RELB
RHOA
RIPK1
RIPK2
SIRT3
SIRT4
SIRT5
SLC2A1
SLC2A1
SLC2A2
SLC2A3
SMAD1
SMAD7
STAT1
STAT1
STAT2
STAT3
STAT3
TAB1
TGM2
TLR1
TLR2
TLR3
TLR4
TLR5
TLR6
TLR7
TLR8
TLR9
TLR10
TP53 (p53)
TRAF2

TRAF6
TYK2
UBE2N
UQCRH
USP7
VISA
